# Stem and Progenitor Cells in Human Cardiopulmonary Development and Regeneration

**DOI:** 10.1155/2017/2653142

**Published:** 2017-09-17

**Authors:** Silvana Bardelli, Marco Moccetti

**Affiliations:** ^1^Swiss Institute for Regenerative Medicine, Foundation for Cardiological Research and Education, Via ai Söi 24, 6807 Taverne, Switzerland; ^2^Cardiology Department, Cardiocentro Ticino Foundation, Via Tesserete 48, 6900 Lugano, Switzerland

## Abstract

Already during embryonic development, the heart and the lung are thoroughly connected organs. Their interdependence allows our survival in the terrestrial environment by coupling cardiac output and gas exchange. The knowledge on developmental processes involving stem and progenitor cells is crucial to understand the onset of human cardiopulmonary diseases. The precise identification of various adult endogenous progenitors is still incomplete. Thus, caution should be exercised on newly available stem cell-based treatments until specific mechanisms of action are disclosed. The objective is to provide in the nearest future feasible and safer cell therapeutics for the complex pathological condition of human cardiopulmonary diseases. In this paper, we highlight the significant knowledge advancement concerning stem and progenitor cells in the cardiopulmonary field: from embryonic development to adult progenitors until early preclinical models for cardiopulmonary regeneration.

## 1. Development of the Cardiopulmonary System: The Contribution of Stem and Progenitor Cells

Adaptation to terrestrial life happened recently in our evolutionary history. As a consequence of this event, the cardiac and the pulmonary systems developed in parallel to allow the coupling of cardiac function and gas exchange in the lung. In mammals, the cooperation of these two systems is already apparent during embryonic development: while the heart tube loops and asymmetrically divides into the mature cardiac chambers, the lung anterior endoderm protrudes into the cardiac embryonic mesoderm. This interdependence forms the cardiopulmonary circulation, a specialized compartment that connects the heart and the lungs: it receives the cardiac output to allow gas exchange and to provide oxygenated blood to the systemic circulation.

Cardiac morphogenesis occurs prior to lung development [1, 2]. The embryonic heart early provides pump function that is fundamental for fetal and postnatal life. Heart development is regulated by highly conserved tissue-specific transcription factors, signaling molecules, and noncoding RNAs. Central to this network are the transcription factors Wnt, NKX2-5, GATA4, and SRF, which, together with their target DNA elements, form an evolutionarily conserved subcircuit essential for development [3]. The process of looping morphogenesis brings the venous pole ventral to the foregut endoderm. This mesoderm-endoderm interaction is crucial to lung development. The embryonic lung evaginates from the anterior endoderm which will form also the trachea and larynx. The organ's epithelium derives from the endoderm, while lung mesenchyme is of mesodermal origin. From the distal region of the laryngotracheal groove [4, 5], embryonic epithelial progenitors divide rapidly and generate sequentially the primary and secondary bronchial airways and the alveolar structures. As a consequence, the adult respiratory tree is formed [6, 7].

The spatially and temporally coordinated development of the embryonic heart and lung raises the possibility of a common multipotent progenitor originating in both organs and their physiologic connection in terrestrial mammals. Recently, Peng et al. [8] reported a novel population of multipotent cardiopulmonary mesoderm progenitors (CPPs) that arises from cardiac posterior pole prior to lung development. Wnt2^+^/Gli1^+^/Isl1^+^ CPPs were identified by lineage tracing and clonal analysis experiments and proved to generate the mesoderm lineages of the cardiac inflow tract, pulmonary vascular and airway smooth muscle, lung proximal endothelium, pericyte-like cells, and also cardiomyocytes. The foregut endoderm that is required to connect the pulmonary vasculature to the heart regulates the development of CPPs through the Sonic Hedgehog (Shh) network. Shh activates its effector Gli1 that is coexpressed with Wnt2 and Isl1 in CPP cells. According to lineage-tracing experiments, the authors observed that Hedgehog signaling is required to direct the development of CPPs towards the lung smooth muscle lineage and initiates the cardiopulmonary connection. The authors reported that the earliest cardiac progenitors that are located in the second heart field are characterized by the expression of Isl1. The Isl1-positive (Isl1^+^) population further subdivides into Isl1^+^/Nkx2.5^+^ cells in the ventral/medial domain and the Isl1^+^/Nkx2.5^−^ subpopulation in the lateral/dorsal domain. This latter subpopulation, characterized only by the expression of Isl1, generates all layers of the lung vasculature and the myocardial inflow tract at E8.5. Specifically, Isl1^+^ progenitor cells generate the ventral lung mesenchyme that connects to the cardiac inflow tract, while Nkx2.5-positive progenitors give rise to the myocardium close to the pulmonary vein. Wnt2-positive progenitors, located exclusively in the posterior pole of the developing heart at E8.5, form cells within the cardiac inflow tract, not within the outflow tract. These cells are the ones that move to the lung bud in its early development. Later during embryonic development, Wnt2^+^ cardiac progenitor cells generate all mesodermal lineages of the heart including cardiomyocytes and the endocardium. Additionally, they give rise to the pulmonary vasculature, lung pericytes, and airway smooth muscle cells in the developing lung. Therefore, the authors demonstrated that Wnt2^+^ cells represent multipotent progenitors in the developing lung and inflow tract of the heart. The authors also reported that the subpopulation of cardiac Gli1-positive cells contributes to the cardiac mesodermal compartment as well as the early lung bud. Overall, the population of Wnt2^+^/Gli1^+^/Isl1^+^ cells generates the majority of mesodermal cells in the cardiac inflow tract and in the lung. Therefore, pulmonary vascular and airway smooth muscle cells, proximal endothelium, and pericyte-like cells derive clonally from these progenitors. Importantly, alterations of this developmental pattern cause congenital defects such as tetralogy of Fallot syndrome in the newborn or persistent pulmonary hypertension. Ultimately, understanding the role of cardiac mesoderm and lung endoderm interaction during development would provide mechanistic insights into the congenital cardiopulmonary diseases where vascular patterning and differentiation are perturbed. Furthermore, deciphering the signaling pathways necessary for pulmonary vascular development could potentially shed light on mechanisms involved in vascular regeneration and remodeling in adult pulmonary diseases. Adult cardiac diseases such as myocardial infarction (MI) result in a massive loss of cardiomyocytes that leads to heart failure. Successful therapies for these diseases are lacking. There is an urgent need to clarify the mechanisms that regulate heart and lung development to design effective approaches for cardiopulmonary regeneration.

## 2. Adult Progenitor Cells in Human Heart and Lung Regeneration

Cardiac and pulmonary diseases are frequent. They impact significantly on healthcare costs. A recurring question in biology is whether regeneration occurs in these adult organs.

In the heart, the focus is whether adult cardiomyocytes (CMs) proliferate and to what extent. This long-debated question raises controversies in the field. In the last decades, human cardiomyocyte proliferation was documented, as well as its steady state. Bergmann et al. [9] presented a study on human CM stereology combined with quantification of genomic ^14^C concentrations in cardiomyocyte nuclei (retrospective birth dating). They reported that, according to the analysis of CM volume and nuclear DNA synthesis, the CM number did not change substantially in postnatal life and remained constant throughout the whole human life span. Specifically, compared to both cardiac endothelial and mesenchymal cells, cardiomyocytes showed the highest extrapolated turnover rate restricted to the first decade of life; cardiomyocyte turnover decreased with age exponentially and was ≤1% in adults. Mollova et al. [10] with the same technique, that is, stereology, found that most postnatally born CMs are generated in young humans: their number increased by 3.4-fold over the first 20 years of life, indicating that the highest cardiac cell proliferation rate occurred in young adults. No consensus exists on the magnitude of adult cardiomyocyte renewal, with estimates ranging from no turnover rate to complete cell exchange in a few year lifespan [11, 12].

Overall, the reported results suggest that the mammalian heart possesses a measurable capacity for renewal. Importantly, intense debate exists concerning the source of the newly generated cardiomyocytes: it is not yet clear whether cardiomyocytes are renewed through differentiation from a stem/progenitor population or through cell division by existing cardiomyocytes [13, 14]. Nevertheless, these two possibilities are not mutually exclusive, and both represent possible opportunities to increase cardiomyocyte generation for cardiac regenerative therapies. In the field of cardiac regeneration, there is a considerable interest in whether transdifferentiation events might generate new cardiomyocytes. Bone marrow-derived cells like hematopoietic stem cells and mesenchymal stem cells [15, 16] were thought to differentiate to cardiac muscle and contribute to functional recovery after MI. However, results from subsequent studies indicate that these cell types may contribute to heart repair by indirect paracrine mechanisms, as opposed to direct differentiation into myocardial cells [17, 18]. The mechanism of cardiomyocyte dedifferentiation might also occur. This process is characterized by a reduction of sarcomere structures and the expression of fetal gene markers. A significant advancement in the field will be to understand how dedifferentiation is initiated and identify the target molecules that induce these phenotypic changes [19, 20].

Based on the ongoing debate on the actual capacity of the adult human heart to renew cardiomyocytes, alternative therapeutic approaches to augment endogenous regeneration are explored such as the administration of stem or progenitor cells to the heart or the stimulation of endogenous cardiac progenitors.

To this extent, however, a clear definition of endogenous cardiac progenitors is necessary. The issue is still elusive and controversial as of today.

Numerous putative adult cardiac progenitors have been characterized by the positivity of different markers. Specifically, in 2003, Oh et al. documented a cardiac progenitor cell based on the expression of murine Sca1 antigen [21]. This population can be enriched for either high efflux of Hoescht dye through an ATP-binding cassette transporter (side population cells) or high expression of PDGFRa. The enriched population shows multilineage potential and differentiation towards cardiomyocytes in vivo. In the same year, Beltrami et al. documented an alternative cardiac progenitor characterized by the expression of the receptor c-kit (CD117, Stem Cell Factor receptor) [22]. In more recent years, Ellison et al. [23] concluded that c-kit-positive cells are necessary and sufficient to regenerate an acute adult myocardial injury based on a cardiotoxic isoproterenol treatment model. In 2004, Messina et al. reported the isolation of adult cardiac progenitors that grow in adherent spheres, named cardiospheres [24]. Cardiospheres are composed of a combination of progenitor cells, cardiac myocyte-like cells, and vascular cells. The authors suggested that these cell types are the progeny of a small subset of undifferentiated cells that express different stem cell markers such as c-kit and Sca-1. Cardiosphere-derived cells are isolated from adult murine and a human heart and can be expanded in vitro for therapeutic use. The identification of multiple progenitors and the concomitant-limited therapeutic regeneration observed in studies performed so far led some investigators to conclude that most progenitors are the same cell at different stages of differentiation [25–29].

The adult mammalian lung is organized into two major compartments: the airways that conduct gases and the alveoli where gas exchange occurs. Approximately 40 different cell types exist within the adult lung. The epithelial lineages are the best defined. Their characterization is based on murine lineage-tracing studies. These studies might reflect the organization of the adult human lung; however, human lung epithelium might possess unique properties.

The steady state lung is a low cellular turnover tissue that includes quiescent stem or progenitor cells. These cells participate in the repair of the damaged lung [30–33]. Basal cells are characterized by a small height compared to adjacent luminal cells, and they are located at the basement membrane [34]. Basal cells express the N-terminus-truncated isoform of TRP63 (p63), cytokeratin 5 (KRT5), nerve growth factor receptor (NGFR), and podoplanin (PDPN) [35]. These cells are self-renewing and multipotent: they generate other basal cells and also secretory and ciliated cells [36]. Recent studies by Pardo-Saganta et al. [37] demonstrated that, under steady state conditions, the basal cell population is heterogeneous: they express activated Notch2 intracellular domain (Notch2ICD) and c-myb (Myb) in secretory and ciliated cells, respectively. Basal cells are located in the murine trachea and bronchi while in humans, they are found more distally, in the small bronchioles.

Secretory or club cells (formerly known as Clara cells) are dome shaped and possess secretory granules in their cytoplasm. Murine secretory cells are self-renewing and differentiate into ciliated cells. These cells are present in the murine trachea, bronchi and bronchioles, and throughout the human airway epithelium. Recent studies by Tata et al. [38] indicated that they are highly heterogeneous.

Ciliated cells are also present throughout the large and small airways. They are characterized by multicilia on their apical surface and are positive for the nuclear transcription factor FoxJ1. Lineage-tracing studies document that they are terminally differentiated cells. Ciliated cells are produced directly from basal cells following injury. Neuroendocrine cells are single cells or organized clusters in close contact with nerve fibers. They are characterized by the expression calcitonin gene-related peptide (CALCA), chromogranin A, and achete-scute homolog 1 (ASCL1). They are present in murine large and small airways and are enriched at the branch points of airways. Pulmonary neuroendocrine cells perform multiple functions such as oxygen sensing and mechanotransduction.

Alveolar epithelial type 2 and type 1 cells are cuboidal surfactant-producing and gas-exchanging cells, respectively. Recent studies through lineage-tracing analysis demonstrated that type 2 cells maintain the homeostatic turnover of type 1 cells and clonally generate more type 2 cells in the adult lung [39]. The zone of transition from the bronchioles to the alveoli is referred to as the bronchioalveolar duct junction (BADJ). Within this region, bronchioalveolar stem cells (BASCs) are present. They were identified based on their proliferation after bleomycin injury [40]. In humans, BASCs have not been clearly characterized.

Interestingly, cellular plasticity is now an emerging concept in the biology of multiple adult organs. Multiple studies recently indicated that in various tissues, cellular plasticity is a common phenomenon in the process of repair after injury [41–43]. In the lung, evidence for plasticity derives from cell ablation experiments. Tata et al. [44] reported that in the tracheal epithelium, fully mature secretory cells dedifferentiated into basal stem cells following diphtheria toxin-induced stem cell ablation. Interestingly, secretory cells started to replicate when over 80% of the basal cells were ablated by the treatment. The signals that regulate cell plasticity are yet to be defined. Tata and Rajagopal reported that transdifferentiation can also occur [45]: fully differentiated neuroendocrine cells in the small airways generate secretory cells as well as ciliated cells following naphthalene-induced injury or after H1N1 influenza-induced injury. Lineage-tracing experiments demonstrate that epithelial stem and progenitor cells maintain a stable identity during steady state conditions but can display remarkable lineage plasticity following injury. In humans, our knowledge on cellular plasticity is preliminary. In vitro results demonstrate the plasticity of human lung epithelial cells. However, the results might not reflect the plasticity observed in living organisms. Further advancement of the concept of cellular plasticity will certainly need confirmation in the next decades.

In the human heart, there is a lack of consensus on the composition of the nonmyocyte cell population. Very interestingly, a recent study by Pinto et al. revealed that fibroblasts represent a relatively minor cell population and that endothelial cells are the most abundant cell type in healthy adult human hearts [46]. The authors used newly available genetic trackers, flow cytometric analysis, and an unsupervised clustering algorithm (SPADE, Spanning-tree Progression Analysis of Density-normalized Events). The analysis showed that approximately 65% of cardiac cells are endothelial cells, 10% are leukocytes, and about 25% are cardiomyocytes. These unexpected results highlight the fact that the cardiac fibroblast population is much smaller than previously reported [47]. Furthermore, a comprehensive understanding of cardiac cellular composition will guide the development of new therapeutics to promote heart repair and regeneration. Overall, these findings redefine the cellular composition of the adult murine and human heart and indicate that the endothelial cell compartment might play a potentially important role in cardiac homeostasis, disease, and regeneration.

## 3. Current Stem Cell-Based Therapeutic Approaches for Cardiopulmonary Diseases

Pulmonary arterial hypertension (PAH) is associated with right ventricular hypertrophy or failure. This is the result of pressure overload in the right ventricle. Current therapeutic approaches are still experimental, and we need to be cautious in stating their efficacy. However, potentiality exists and current treatment options might expand in the next decades.

Overall, stem and progenitor cell therapy in cardiopulmonary diseases demonstrates to be effective in animal models of PAH. Mainly, these stem cell-based experimental models lay on the observation that stem and progenitor cells might regenerate pulmonary vasculature. Accordingly, endothelial progenitor cells (EPCs) are good candidates towards this goal: endothelial progenitors are circulating cells derived from the bone marrow. They are able to differentiate into mature endothelial cells to repair the vasculature. It is still not clear how endothelial stem or progenitor cells exert their effect when administered to the lung. Proper engraftment in the lung tissue is thought to happen rarely. A combination of concomitant biological mechanisms is more likely to occur, including stem cell-induced paracrine effect due to the release of microvesicles or exosomes. Noncoding microRNAs are more recent players in this field. Interestingly, Spees et al. investigated the effect of monocrotalin (MCT) on the engraftment and differentiation of GFP-positive bone marrow-derived cells in rodent models of PAH [48]. The authors observed the engraftment of the administered cells in the lungs and their differentiation into pulmonary epithelial cells (Clara cells), vascular endothelial cells, and smooth muscle cells. Furthermore, GFP-positive cells engrafted in both the right and the left ventricles of hyperthophic rat hearts. In the right ventricles, administered cells differentiated mainly into vascular cells and cardiomyocytes. No cell fusion events were observed between endogenous cardiac cells and administered bone marrow-derived cells. Combination therapy including the administration of stem or progenitor cells together with pharmacological agents is in general more effective. Sun et al. administered cilostazol, a phosphodiesterase III inhibitor, together with EPCs three days after MCT injection [49]. The authors observed reduced remodeling of pulmonary resistance arteries resulting from proliferation of endothelial cells and vascular smooth muscle cells. In general, combination therapy was more successful than EPCs or citostazol alone in preventing vascular remodeling due to MCF-induced PAH.

Takemiya et al. observed that intravenous administration of mesenchymal stem cells (MSCs) in rat lungs affected by MCT-induced PAH was not sufficient to lower pulmonary artery pressure. However, when MSCs were delivered in combination with prostacyclin synthase, the authors reported a significant decrease in pulmonary artery systolic pressure and right ventricular dilation. Notably, paracrine effect due to cell-mediated release of soluble factors rather than massive cell engraftment is thought to exert the effects observed.

In the clinical condition of emphysema, the alveolar epithelium is damaged and repair processes are unlikely to occur. The role of all transretinoic acid (ATRA) is currently under investigation in the therapeutic treatment of emphysema. Retinoic acid is the active metabolite of vitamin A (i.e., retinol) that is essential for multiple cellular functions such as cell homeostasis and differentiation. Retinoic acid is acquired from diet. However, the long-term use of oral retinoic acid causes side effects such as dry skin, headache, hyperlipidemia, muscle, and bone soreness. Specifically, Mao et al. performed a double-blind, placebo-controlled feasibility trial to test the long-term administration of ATRA. Patients affected by moderate to severe emphysema were subjected to the standard of care plus twice-daily oral administration of ATRA for 12 weeks [50]. The study did not show any therapeutic effect on emphysema, and side effects were observed. Brooks et al. tested the effect of aerosolized ATRA in rodent models of emphysema and demonstrated that it is feasible and represents a safer alternative to oral retinoic acid [51].

The processes involved in lung epithelial repair are currently unknown despite the significant advances in stem cell research over the past decades [52–54].

Personalized medicine approaches are essential for the treatment of cystic fibrosis. Over 1500 known mutations of the CFTR (cystic fibrosis transmembrane conductance regulator) gene exist. Each of them results in distinct functional pathologic variables. CFTR is expressed on the surface of plasma membranes, specifically in ciliated cells. It is a chloride channel that, when alterated, produces impaired chloride and bicarbonate secretion resulting in thicker mucus and recurrent infections. The generation of patient-specific in vitro models for this clinical condition is crucial. Culture of patient-derived primary human airway or nasal epithelial cells and their targeted differentiation may constitute a valuable objective of therapeutic investigation in this field. Alternatively, the differentiation of patient-specific-induced pluripotent stem cells (iPSCs) into adult epithelial cells might be pursued. Patient-specific stem or progenitor cell treatments in preclinical models of cystic fibrosis will thus allow drug development in the future [55].

Notably, the current position of the COPD Foundation (https://www.copdfoundation.org) on stem cell therapy is cautious. The foundation warns on several clinics providing alleged stem cell-based treatments for incurable lung diseases, including chronic obstructive pulmonary disease (COPD). FDA did not approve such treatments. Therefore, the COPD Foundation does not recommend the use of autologous stem cell therapy for the treatment of COPD or other lung diseases until more convincing proof of effectiveness is provided. The COPD Foundation encourages patients to participate in the clinical trial that tests the development and potential benefit of this approach.

Overall, stem cell-based therapeutic approaches on human cardiopulmonary diseases are still at their preliminary stage. We acquired valuable information of endogenous cardiac and pulmonary stem or progenitor cells that are distributed in different compartments of these organs. Stem and progenitor cells may represent key protagonists of newly available treatments. The knowledge we acquired so far, although insufficient to guarantee an immediate therapeutic use, warrants further studies to impact on this massive clinical demand.

## 4. Reflections on Current Therapeutic Developments

As mentioned earlier, no consensus exists so far on the characterization of endogenous pulmonary and cardiac stem or progenitor cells. Many authors raise the possibility that the same cell at subsequent differentiation stages was characterized by different groups.

Once properly identified, specific progenitors might be successfully employed in lineage-tracing studies to understand their role in animal models of disease. Furthermore, specific sorting of surface markers through fluorescence-activated cell sorter (FACS) might be used to enrich candidate progenitor cells more homogeneously. This targeted approach will shed light on the specific role of the sorted cells when administered in vivo.

Administration of endothelial progenitor cells (EPCs) or mesenchymal stromal cells (MSCs) is the emerging strategy for the treatment of severe cardiopulmonary diseases such as pulmonary arterial hypertension. These studies are preliminary and rely mainly on preclinical animal models. MSCs are thought to exert their effect through immunomodulatory properties. Nevertheless, the precise mechanisms that allow stem or progenitor cells to act in cardiopulmonary remodeling are still unknown. Possibly, multiple concomitant biological, biochemical, and biomolecular cues are involved.

Induced pluripotent stem cells (iPSCs) represent an additional cell source. They are patient specific and might potentially serve as a renewable source. The immediate impact of iPSC technology does not lie in regenerative medicine applications but mainly in the study of the cellular mechanisms that generate cardiopulmonary diseases. This allows potential patient-specific drug screening and future gene therapy, a powerful approach within the field of personalized medicine.

On the other hand, increasing knowledge on the mechanisms that control embryonic cardiopulmonary development might highlight key molecular effectors. The same pathways are frequently impaired at the onset of cardiopulmonary diseases. Additionally, the recent identification of a common progenitor cell that directs development of the cardiopulmonary circulation further strengthens the connection of these two organs.

## 5. Conclusions

Accumulated knowledge in preclinical models and in preliminary clinical trials suggests that stem cell-based therapies may represent potential strategies for cardiopulmonary repair after injury. In parallel, further characterization of endogenous stem and progenitor cells in the lung and in the heart provides a sound scientific basis for therapeutic use in cardiopulmonary diseases. This approach lies on the precise identification of specific markers for each progenitor cell type.

Remarkable advances of basic research on human cardiac and pulmonary stem cells in the past decades have sustained the submission of numerous investigational new drug applications for clinical trials in humans. Although the current understanding is still limited to guarantee a safe human application for cardiopulmonary diseases, autologous stem and progenitor cells are emerging as key players for newly available therapies. The nearest future will hold better insights to develop safer and feasible therapeutic options. This further advancement will happen only if a scientifically sound approach leads the studies of human cardiopulmonary diseases that still constitute an area of unmet clinical need.
